# Research and application of artificial intelligence in dentistry from lower-middle income countries – a scoping review

**DOI:** 10.1186/s12903-024-03970-y

**Published:** 2024-02-12

**Authors:** Fahad Umer, Samira Adnan, Abhishek Lal

**Affiliations:** 1 Department of Surgery, Section of Dentistry, The Aga Khan University, Karachi, Pakistan; 2 Department of Operative Dentistry, Sindh Institute of Oral Health Sciences, Jinnah Sindh Medical University, Karachi, Pakistan; 3 Department of Medicine, Section of Gastroenterology, The Aga Khan University, Karachi, Pakistan

**Keywords:** Artificial intelligence, Dentistry, Deep learning, Machine learning

## Abstract

Artificial intelligence (AI) has been integrated into dentistry for improvement of current dental practice. While many studies have explored the utilization of AI in various fields, the potential of AI in dentistry, particularly in low-middle income countries (LMICs) remains understudied. This scoping review aimed to study the existing literature on the applications of artificial intelligence in dentistry in low-middle income countries. A comprehensive search strategy was applied utilizing three major databases: PubMed, Scopus, and EBSCO Dentistry & Oral Sciences Source. The search strategy included keywords related to AI, Dentistry, and LMICs. The initial search yielded a total of 1587, out of which 25 articles were included in this review. Our findings demonstrated that limited studies have been carried out in LMICs in terms of AI and dentistry. Most of the studies were related to Orthodontics. In addition gaps in literature were noted such as cost utility and patient experience were not mentioned in the included studies.

**Supplementary Information:**

The online version contains supplementary material available at 10.1186/s12903-024-03970-y.

## What is already known

Artificial Intelligence is being used extensively in high-income countries in different sectors including healthcare.

## What this study adds

The scarcity of the current application and research of artificial intelligence in dentistry in low-middle income countries.

## How this study might affect research, practice, or policy

Application and research in artificial intelligence pertinent to dentistry in low-middle income countries can greatly enhance practices and policies leading to better patient care.

## Introduction

Artificial Intelligence (AI) techniques such as *Machine Learning (ML) and Deep Learning (DL),* have the potential to execute complex diagnostic tasks, currently performed by dental specialists, that leads to improved diagnostic accuracy and increased efficiency. Adequately trained AI has the ability to detect teeth, identify pathologies and anomalies including dental caries, missing or lost teeth, periapical lesions, and maxillofacial abnormalities from dental radiographs without the involvement of any dentist or specialist in significantly less time and with more accuracy [1–3].

However, AI research and application in real life scenarios is a resource-intensive task and relies upon both supervised and unsupervised learning techniques that require properly curated and annotated high quality datasets. These specific types of datasets are difficult to procure and compile.

In addition, AI research in health care warrants specialized human resources including medical professionals who are familiar with AI terminologies, norms, and standards. These domains are not a part of any undergraduate or postgraduate at the moment curriculum and require additional specialized training and expertise [4, 5]. Finally, AI researchers need to have access to expensive hardware such as graphic processing units (GPU) which are difficult to acquire in a resource capped environment.

A combination of all these factors may lead to limited advancement in health care AI research and its utility in Low and Middle-Income Countries (LMICs) [5, 6] Ironically, it is in these same regions where the use of AI may have more value in terms of support and standardization of clinical judgment through data-orientated approaches [7]. Global dental health is complex and encompasses numerous hidden variables, including differences in disease prevalence between various races and ethnicities based on genetics. Therefore, the training datasets in dentistry generated from high-income countries would not accurately represent the population, features and disease patterns for LMICs. AI trained on datasets from high-income countries may thus introduce errors if applied in a differently placed population groups [8]. Failure to tune and align the model to a particular population could give rise to certain unintended consequences such as affecting fairness, introducing biases, and disrupting the appropriateness of that algorithm [9]. Therefore, it is quintessential that AI algorithms be trained upon context-specific environment, establishing their relevance and application [8].

Low-middle income countries face many challenges in terms of healthcare access and resources, especially dentistry. Investigating the role of AI in such countries relevant to dentistry can help identify the current applications and gaps to enhance and improve access of dental care. To optimise AI’s impact and relevance, it is essential to comprehend how it might be used for innovation in oral healthcare in settings with limited resources.

In order to identify the utility and development of AI pertaining to dentistry in LMICs, this scoping review intended to include all relevant scientific publications to analyze various study characteristics, with particular focus on the origin and quality of datasets used as well as any challenges linked to carrying out AI based research in LMICs. The level of maturity and integration of AI models mentioned were also be analyzed. In addition, considerations of cost-effectiveness or cost-utility analyses were identified. Any AI application having a patient interface or interaction was evaluated for recording patient experience in this regard.

## Methods

This scoping review was conducted using a predetermined protocol following the PRISMA Extension for Scoping Reviews guidelines [10]. The protocol can be accessed through the Open Science Framework platform (https://osf.io/t62k3/).

### Search strategy

A 3-prong search strategy defining technique of interest (AI, DL, ML), the specialty of interest (Dentistry), and setting of interest (LMICs) was developed by the authors in collaboration with a medical information specialist (Librarian, Aga Khan University Hospital, Pakistan). An LMIC was defined according to World Bank Group Classification of Economies. The studies included in this scoping review were based on the criteria of low-middle income countries as set by World Bank [11].

(World Bank Country and Lending Groups – World Bank Data Help Desk. https://datahelpdesk.worldbank.org/knowledgebase/articles/906519-world-bank-country-and-lending-groups).

The authors conducted a pilot search based on various combinations of key search terms to formulate the final search strategy which was subsequently used in this review.

### Literature search

A comprehensive literature search was conducted from Jan 2010 to Feb 2023 to identify relevant publications in three major health sciences databases i.e., PubMed, Scopus, and EBSCO Dentistry & Oral Sciences Source. Furthermore, a manual search was done by the authors in Google Scholar and IEEE Xplore databases to identify relevant literature not present in the aforementioned databases.

### Search terms

The search terms used for this purpose were; “Dentistry” OR “Dental AND Artificial Intelligence” OR “Deep Learning” OR “Machine Learning” AND “LMIC” OR “Low Resource setting” OR “Low and Middle Income” OR “Underdeveloped Nations” OR “Developing Countries” OR “Developing nations” OR “Economically Developing Countries” OR “Economically Developing Nations” OR “Emergent Nations”.

All primary quantitative and/or qualitative research pertaining to the implementation of AI in the context of dental health conducted in any country defined as LMIC, published in a peer reviewed journal, in English language, and published after 2010 were included. Any published protocols, conference proceedings, letters to editor, and policy documents were excluded.

### Screening process

Selected citations were exported to Endnote version 20.0 (Clarivate Analytics) and duplicates were deleted. The title of each article was screened in accordance to the preset inclusion/exclusion criteria by (F.U, S. A and A.L). Any disagreement in this regard was resolved by discussion between the authors. The data from the selected studies was extracted on a predetermined proforma to chart characteristics of the included studies and key findings. These included country of origin, field of dentistry where AI was applied, dataset types and sources, annotation of dataset, description of annotators, algorithms used, computational resource, cost utility analysis, and challenges of conducting AI centered research in LMICs, if mentioned.

### Eligibility criteria

The following inclusion criteria was applied:

1. Primary studies
2. Studies related to dentistry
3. Studies utilizing artificial intelligence models such as machine learning and deep learning.
4. Studies published till date

The following exclusion criteria was applied:

1. Reviews, editorials, commentaries, and conference papers
2. Studies published in languages other than English
3. Non indexed studies

### Data synthesis

In this scoping review, we have attempted to provide a comprehensive overview of the included studies. This involved examining various relevant aspects such as the origin of research, source of datasets, study designs, datasets curation and annotation, performance metrics, and the maturity of AI algorithms used. However, it should be noted that these studies varied significantly in terms of their focus of research question/task, type of input data, model architecture, and other factors of interest. As a result, we conducted a narrative synthesis to effectively summarize the findings and provide insight into the current state pertinent to AI in LMICs.

## Results

**Fig. 1:**
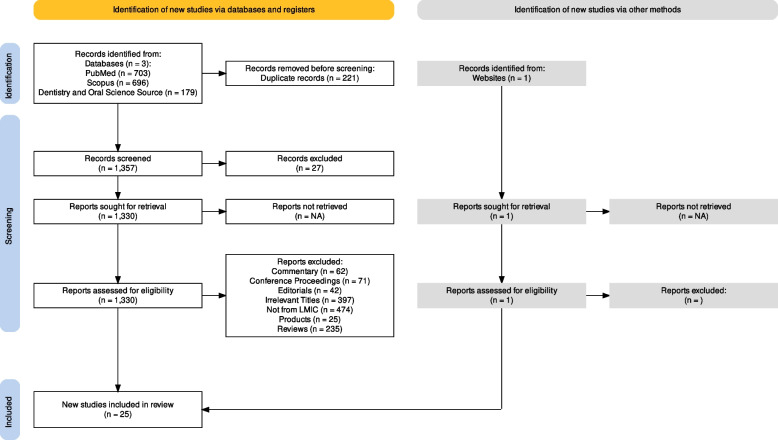
PRISMA flow chart

A total of 1578 articles were initially identified following a detailed search using electronic and manual literature search platforms. After the removal of duplicates, 1357 articles underwent further screening. 27 articles were excluded on the basis of irrelevancy. A total of 1330 studies underwent a screening process for titles and abstracts. Conference proceedings, commentaries, editorials, irrelevant titles, studies not from LMICs, description of products, and reviews were excluded. Additionally, one study obtained from gray literature through Google Scholar was identified by using relevant search terms. After thoroughly scrutinizing the studies, 25 articles fulfilled the eligibility criteria. Hence, these articles were subjected to final analysis. The screening process used for this study is presented in the PRISMA flow chart (Fig. 1).

The overall characteristics of the included studies as extracted on the specially designed data extraction form are given in Table 1.

**Table 1:** Summary characteristics of the studies

	Authors	Country	Specialty	Study design	Study theme	Dataset Source	Datatype	No. of annotators	Annotators specialty	Sample size	Task	Types of Neural networks/ Models/algorithms	Performance metrics	Maturity
1	Talaat et al	Egypt [12]	Orthodontics	Validation study	Classification	Local	Intraoral photographs	2	Both orthodontists	700	Malocclusion detection	CNN (YOLO)	Accuracy: 99.99 Preceison:0.99 Recall:1 F1 score:1	2
2	Khazaei et al	Iran [13]	Orthodontics	Validation Study	Classification	Local	Lateral Cephalograms	N/A As sex was input from patient records	NM	1476	Gender determination	CNN, Dense Net, ResNet, VGG	DenseNet Accuracy 0.9 Resnet Accuracy 0.62 VGG 16 Accuracy 0.75	2
3	Ehtesham et al	Iran [14]	Oral Medicine	Pilot study	Decision Support System	Local	Health records	N/A As the cases and symptoms were retrieved from medical records	NM	500 training cases, 39 testing cases	Differential diagnosis	ML (Nearest neighbor algorithm)	Metrics were not mentioned explicitly but out of 39 diagnosis there were 9 misdiagnosis	4
4	Koopaie et al	Iran [15]	Restorative Dentistry	Case-control study	Classification	Local	Health records + cystatin S levels of saliva	3	Dentist	20	Caries detection	ML, ANN, XGBoost Random Forest SVM	ANN: Acc 90%, Sen 100%, Spec 72%. XGboost: Acc 89%, Sen 93%, Spec 84%. Random Forest: Acc 85%, Sen 93%, Spec 76%. SVM: Acc 85%, Sen 86%, Spec 84%.	2
5	Adnan et al	Pakistan [1]	Endodontics	Validation study	Instance Segmentation	Local	OPGs	2	Endodontist	40 OPG, 1280 teeth crops	Teeth detection and numbering	CNN	88.2	2
6	Mariam et al	Pakistan [16]	Oral Pathology	Validation study	Diagnostic test accuracy	Local	Histology patches	2	Pathologist	280	Gaze pattern for annotation (Keratin pearl)	Faster R-CNN with Inceptionv2 architecture [2] YOLOv3 with DarkNet53 architecture [3] YOLOv5 with DarkNet53 architecture.	85% less time was consumed in gaze annotations	3
7	Fatima et al	Pakistan [17]	Endodontics	Validation study	Classification and Decision Support System	Local and International	Periapical x rays	2	Radiologist and Dentist	516	Lesion detection Length measurement/ apical foramen Root morphology Root Fracture Case difficulty	CNN Mask RCNN with back bone Resnet 50 Resnet 101 Mobile Net V2 Proposed Backbone Network	Resnet 50: Pre.82, Rec.83, F1.78 Resnet 101: Pre .81, Rec .84,F1 .74 Mobile net V2: Pre.86, Rec .87,F1 .84 Proposed backbone network: Pre 0.86, Rec 0.89, F1 0.89	2
8	Bharathi et al	India [18]	Oral Pathology	Experimental study (why)	Classification	Others (Internet sources, mobile cameras)	Focused photographs	NM	NM	500 total, 140 diabetic	Diabetes tongue	CNN	83	2
9	Patil e tal	India [19]	Oral and Maxillofacial Surgery	Validation study	Classification	Local	OPGs	1	Oral and Maxillofacial surgeon	509	Gender determination (Mandible)	ANN and Regression	The discriminant analysis had an overall accuracy of 69.1%, logistic regression showed an accuracy of 69.9%, and ANN exhibited a higher accuracy of 75%.	2
10	Prasad et al	India [20]	Orthodontics	Validation study	Decision Support System	Local	OPGs, Dental cast, dental clinical data	10–15	Orthodontists	700 training set: test set 70:30 10–15	Treatment planning (Maxilla, mandible, facial landmarks, age, dentition)	ML predictive models: XGB Classifier Random Forest Classifier Linear SVM Decision Tree Classifier Logistic Regression K-Neighbor Classifier Naive Bayes Classifier	The model showed an overall average accuracy of 84%, with the Decision Tree, Random Forest and XGB classifier algorithms showing the highest accuracy ranging from 87 to 93%.	2
11	Katyal et al	India [21]	Orthodontics	Validation study	Decision Support System	Local	OPGs	NM	WebCeph	25	Cephalometric analysis (Maxilla, mandible, facial landmarks, dentition)	CNN	No statistically sig difference between manual tracing, digital tracing and WebCeph, WebCeph was more efficient	3
12	Sherly et al	India [22]	Restorative dentistry	Validation study	Classification	NM	OPGs, Clinical pictures	NM	NM	NM	Caries detection	DL (MDP (maximum directional pattern) using CNN	98.6%	2
13	Benakatti et al	India [23]	Oral and Maxillofacial surgery	Validation study	Identification	Local	OPGs	NM	NM	NM (only mentioned 80% of data set for training and 20% for testing)	Implant system identification	Support vector machine (SVM), K-Nearest Neighbor (KNN), XG boost, and Logistic Regression Classifiers	Best accuracy: Logistic Regression, Overall 0.67	2
14		India [24]	Orthodontics	Validation study	Decision Support System	Local	Dental clinical data	1	NM	1000	Influence of anatomical risk factors in determining number of traumatized teeth per affected individual	MLP model of ANN	Overbite (100%) as the strongest risk factor for TDI in number of teeth of affected individual	2
15	Yadalam et al	India [25]	Oral and Maxillofacial surgery	Validation study	Decision Support System	Local	Dental clinical data	NM	NM	1032,825 (80%) training set, 207 (20%) data authentication, 45 controls	Prediction of postoperative pain after implant surgery (VAS)	ML (Multiple Linear Regression MLR) model	The data set’s output is evaluated using a confusion matrix: the higher the confusion matrix’s diagonal values, the more correct the predictions. The training sample accuracy and the validation sample	2
16	Shwewood et al	India [26]	Endodontics	Validation	Identification and classification	Local	CBCT	2	Endodontists	135, 100 for training and 35 for testing	identification of C-shaped root canal anatomy	DL/CNN (U-Net, Residual U-Net and Xception U-Net)	Xception U-Net with CLAHE provided the best results the mean sensitivity values were highest for Xception U-Net (0.786 ± 0.0378, diagnostic test grade: 78.6% [good]) followed by residual U-Net (mean sensitivity 5 0.746 ± 0.0391, diagnostic test grade: 74.6% [good]) and U-Net (0.720 ± 0.0495, diagnostic test grade: 72.0% [good]). The mean positive predictive	2
17	Mallishery et al	India [27]	Endodontics	Validation study	Decision Support System	Local	Radiovisographs, AAE Case difficulty Assessment forms	2	Endodontists	500	Difficulty of endodontic case	ML (support vector machine (SVM), and deep neural network (DNN))	94.96%	2
18	Moidu et al	India [28]	Endodontics	Validation study	Classification	Local	Periapical x-ray	3	Endodontists	3000 periapical root areas (PRA) on 1950 digital IOPA 450 PRA for model validation 540 PRA on 250 IOPA for testing performance	Periapical lesion	DL (CNN YOLO.v3)	Across all PAI score, 303 PRA (56.11%) exhibited true prediction. **True prediction:** PAI score 1 90.9% PAI scores 2 and 5: 30% PAI scores 3: 60% PAI score 4: 71% **False** **Prediction:** PAI score 2 was interpreted as PAI score 3 (32%) and PAI score 1 (25%). PAI score 5 was interpreted as PAI score 4 (35.8%) and PAI score 3 (20.8%) PAI scores were dichotomized as healthy (PAI scores 1 and 2) and diseased (PAI scores 3, 4, and 5), the model achieved a true prediction of 76.6 and 92%, respectively The CNN model achieved a 92.1% sensitivity, 76% specificity, 86.4% PPV and 86.1% NPV Accuracy:86.3% F1 score: 0.89 Matthews correlation coefficient: 0.71	2
19	Ghosh et al	India [29]	Restorative dentistry	Randomized parallel group study	Dental practice management	Local	Others (Scientific papers)	NM	NM	250	Improving patient recall rate (Patient recall)	topic modeling using Labeled LDA (Latent Dirichlet Allocation)	improved the patient recall rate from 21.1 to 37.8% (*p*-value = 0.024).	4
20	Fidya et al	Indonesia [30]	Orthodontics	Validation study	Classification	NM	Casts	NM	Health records	150	Gender Determination	ML Naive Bayes Decision tree MLP	The accuracy rate of the Naive Bayes method was 82%, while that of the decision tree and MLP amounted to 84%.	2
21	Widyaningrum et al	Indonesia [31]	Periodontics	Validation study	Classification	Local	OPGs	2	Periodontist and General Dentist	1100	Periodontitis	CNN U-Net, RCNN	U-Net showed the characteristic of semantic segmentation, and Mask R-CNN performed instance segmentation with accuracy, precision, recall, and F1-score values of 95, 85.6, 88.2%, and 86.6%,	2
22	Mahto et al	Nepal [32]	Orthodontics	Validation study	Decision Support System	local	Lateral Cephalograms	NM	WebCeph	30	compare the linear and angular cephalometric measurements obtained from web-based fully automated Artificial Intelligence (AI) driven platform “WebCeph”™ with that from manual tracing	CNN	overall Intraclass correlation coefficient for manual and WebCeph tracing was > 0.9	3
23	Thahn et al	Vietnam [33]	Restorative dentistry	Validation study	Classification	Local	Dental clinical data	1	Dentist	1902	Dental caries	CNN Faster Region-Based Convolutional Neural Networks (Faster R-CNNs), You Only Look Once version 3 (YOLOv3), RetinaNet, and Single-Shot Multi-Box Detector (SSD)	For cavitated caries: YOLOv3:87.4% and Faster R-CNN: 71.4% For visually non-cavitated: YOLOv3: 36.9% and Faster R-CNN: 26% Specificity of all 4 models for cavitated caries: 86% Sensitivity of all 4 models for non-cavitated caries: 71%	2
24	Tuan et al	Vietnam [34]	Oral Radiology	Validation study	Segmentation	NM	OPGs	NM	NM	56	new semi-supervised fuzzy clustering algorithm named as SSFC-FS based on Interactive Fuzzy Satisficing for the dental X-ray image segmentation problem.	ML	Not mentioned	1
25	Ngoc et almachine	Vietnam [35]	Endodontics	Validation study	Classification	NM	Bitewing	2	Dentist	130	Periapical lesion diagnosis	CNN Faster R-CNN	Specificity: 97.9% Sensitivity: 89.5% Accuracy: 95.6%	2

**Fig. 2:**
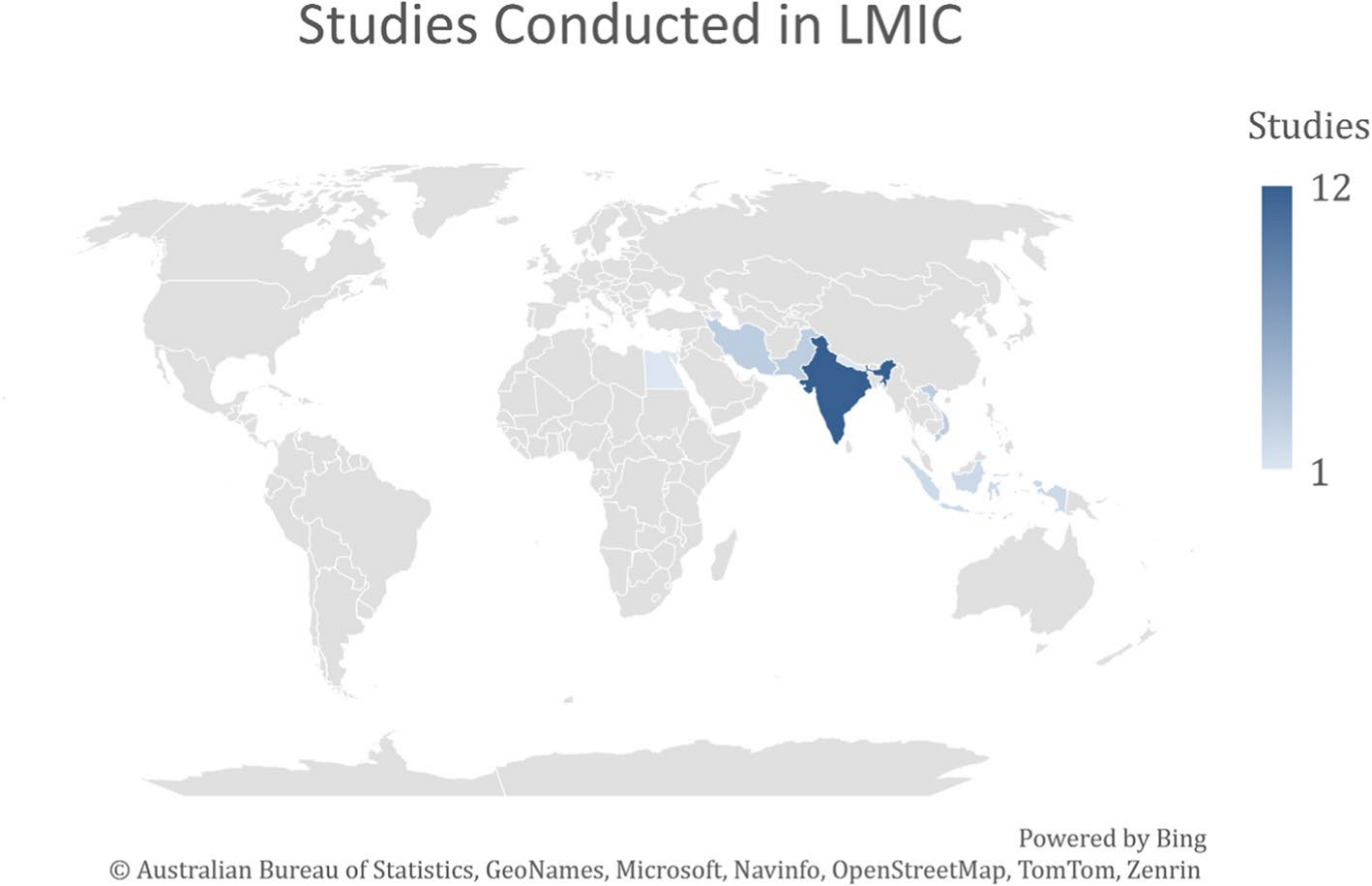
Low-middle income countries where AI has been utilized

Most of the studies were from India (*n* = 12, 48%), followed by Pakistan (*n* = 3, 12%) and Iran (*n* = 3, 12%), as illustrated in Fig. 2.

The majority of studies from India were focused on prediction (*n* = 14, 56%), particularly for gender, as well as identification of pathologies such as dental caries and periapical lesions.

Overall, Orthodontics was the specialty where AI was mostly applied (*n* = 7, 28%), followed by Endodontics (*n* = 6, 24%). The majority of studies were based on validation (*n* = 22, 88%), and those utilizing a quantitative approach (*n* = 20, 80%). The highest number of studies focused on diagnostic test accuracy (*n* = 14, 56%), followed by prediction (*n* = 5, 20%) and identification of various entities (*n* = 3, 12%). The most frequently used type of dataset was Orthopantomograms (OPGs), used singularly or in combination with other types of data modalities. Most studies used local datasets for AI algorithm generation or for model training (*n* = 19, 76%).

Based on the stages of clinical AI development maturity, it was seen that most of the studies (n = 19, 76%) reached stage 2, where the authors had trained and tested AI models on actual clinical datasets. Only one study was at stage 4, where the developed model for achieving differential diagnosis was tested in the actual clinical setting.

Regarding limitations faced by the authors of the included studies, none were mentioned particularly in the context of difficulties or hurdles in carrying out the research in a LMIC setup. The limitations were generic, being confined to the size of the dataset and hence with subsequent limitations related to generalizability of the results. There was also no mention of cost-utility analysis in any of the included studies.

### Heat map

**Fig. 3:**
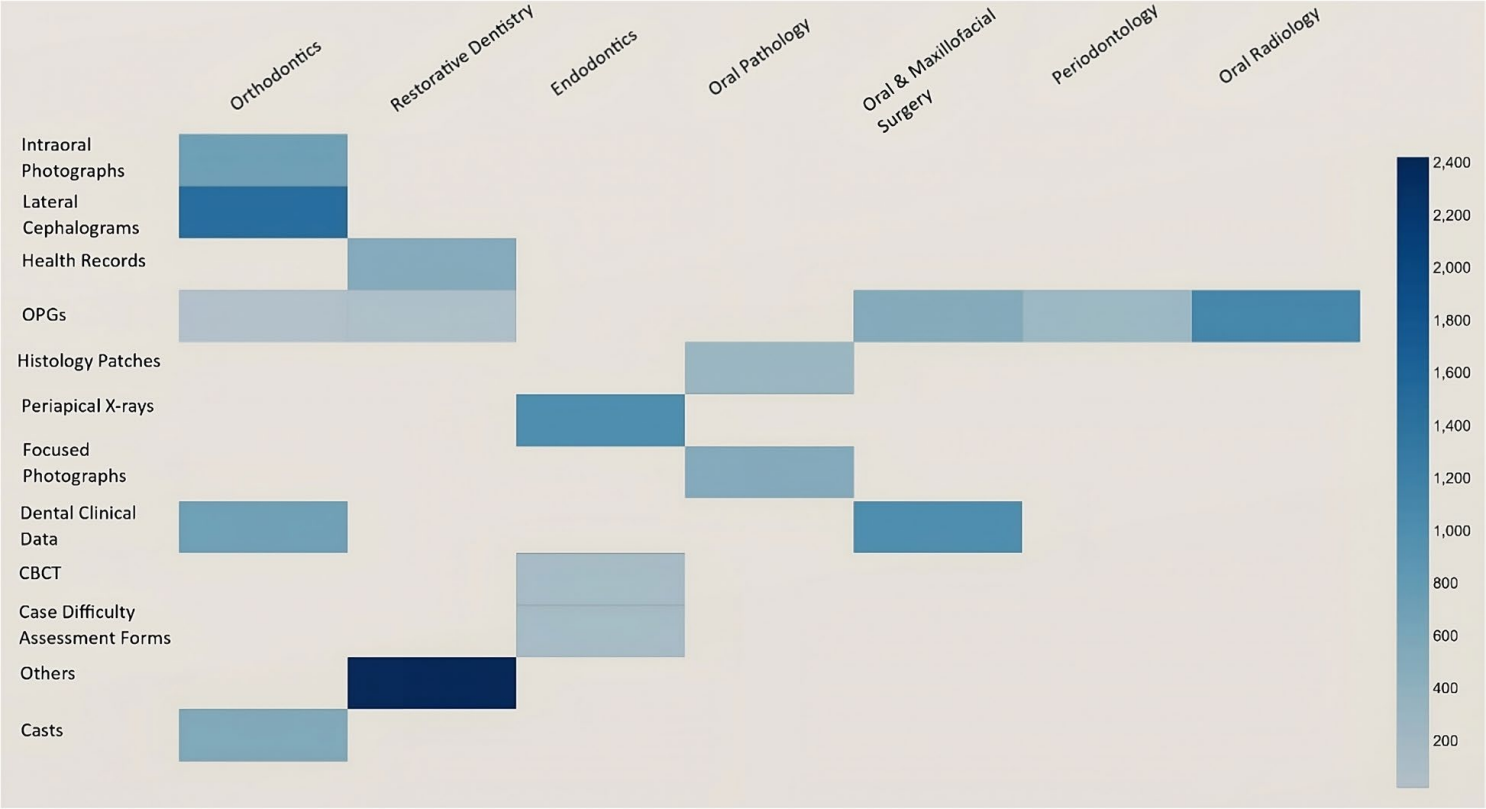
Heatmap of the included studies depicting datasets, specialties, and sample size

The heat map generated for the type of data sets used in the included studies (Fig. 3) against the dental specialties where AI was applied indicated that the highest number of data sets used in Restorative Dentistry were those that were classified as ‘others’. These included health records, Cystatin S levels of saliva, histology patches as well as scientific papers. Lateral Cephalograms and intra-oral photographs were only used as datasets in the specialty of Orthodontics. Orthopantomograms (OPGs) were the most frequently used datasets utilized across specialties including Orthodontics, Operative Dentistry, Oral and Maxillofacial Surgery, Periodontology, and Oral radiology, with the highest number of this type of dataset used in Oral Radiology. For Endodontics, the datasets used were periapical radiographs, followed by CBCT and case difficulty assessment forms. The specialty of Orthodontics overall used a greater variety of datasets including intra-oral photographs, lateral cephalograms, OPGs, dental clinic data, and casts. This indicates that the specialists in this field are trying to develop AI applications based on a multitude of diagnostic and assessment tools.

## Discussion

AI is increasingly being adopted in dentistry in recent times, with particular focus on research in this domain [36]. However, even though AI can be considered a global phenomenon, there has been a dearth in identifying the prospects and contribution of researchers from developing countries in this field of innovation. This omission could potentially lead to biases and health inequities if AI perspectives of underdeveloped nations are not considered, where application potential may be far greater than in developed nations [37]. AI based entities could help provide patients a diagnosis, predict the spread of disease and outbreak predictions when applied in the healthcare sector within LMICs [4]. However, since there was limited reporting of its application in dentistry, therefore, this review was designed in order to encompass the focus and progress of research pertaining to dentistry and AI contributed by low and middle income countries (LMICs). A comprehensive compilation of data from these studies could be generated, highlighting dental specialties which are evolving through the use of AI, domains of research, types, and maturity of AI applications as well as recognize gaps in research. Any challenges put forth by researchers relevant to the development and application of AI would be analyzed, providing evidence for the support and progression of AI based dentistry in LMICs through relevant global agencies. This will enable researchers to focus on these gaps and improve the inclusivity and diversity of AI research in dentistry.

The LMICs category consists of 54 countries, as reported by the World Bank (https://data.worldbank.org/country/XN). Despite our best efforts, only 25 studies from eight LMICs that met our inclusion criteria could be found. This number is significantly lower than the 168 studies found in the most recent comprehensive scoping review, which conducted its search in May 2021 [36]. It is important to acknowledge that the low number of included studies our review is consistent with the broader trend in the healthcare industry, where only 1–3% of research involves investigators from LMICs [38].

Most of the included studies were from India. This observation is consistent with the pattern of AI research in other healthcare domains, where Indian researchers have made a significant contribution [39].

The heat map indicated that Orthodontics and Endodontics were the most common specialties where AI was being applied in LMICs, with dental caries and periapical lesions being the most frequently studied pathologies. The studies mainly used validation methodology with a quantitative approach, and focused diagnostic test accuracy, which is an important domain for LMICs, since accurate and timely diagnosis could result is significant decrease in morbidity, mortality, and expenditure in a resource deprived area. The most commonly used type of data set was orthopantomograms (OPGs), used alone or in combination with other types of data sets. This finding is also in accordance with other scoping reviews [40].

The prevalence of deep learning techniques, specifically Convolutional Neural Networks (CNNs), was found to be prominent in AI algorithms, with a diverse range of CNN architectures being utilized. This dominance could be because CNNs are highly effective in addressing computer vision tasks. Classical machine learning approaches were also observed to be widely adopted, primarily in developing decision support systems, and are consistent with other literature [3, 41]. Furthermore, datasets used in the included studies were relatively small and not representative of the broader population. The small dataset sizes used could be because most studies did not report sample size calculations. Without proper sample size estimations, researchers may not have had a clear understanding of the required number of datasets or diagnostic documentation needed to adequately represent the target population [42]. This lack of planning may have resulted in smaller datasets being used, which can impact the statistical power and generalizability of the results [16, 42]. This challenge is not unique to dental artificial intelligence (AI) research in LMICs, rather it has been widely documented in dental studies even from developed and resource rich countries [5, 36, 43, 44]. Poor quality and deficient datasets can lead to problems such as biases in AI algorithms and may impact the accuracy and reliability of AI in dentistry. The lack of quantity and quality of datasets from LMICs impedes their generalizability, even for these countries, let alone for use in high-income countries or regions with different demographics.

In addition, the curation and annotation methods of datasets used in the included studies were not consistently reported. To prevent biases and inaccuracies in AI algorithms, it is essential to train them using context-specific environments. The above narrative suggests that the datasets used in AI research in dentistry are relatively small, not representative of the broader population, and the reporting of curation and annotation methods of these datasets was deficient. These issues are particularly prevalent in LMICs. This lack of transparency raises questions about the quality and reliability of the datasets, which are critical for development and validation of AI algorithms in dentistry [3, 36, 45]. Therefore, it is important for researchers in LMICs to focus on sourcing high-quality, representative datasets, curation, and annotation methods to improve the development and validation of AI algorithms in dentistry, even though it be challenging and time-consuming in a resource-limited setting.

Without simultaneously addressing the fundamental issue of inadequate infrastructure, the potential of these technologies in global health remains uncertain [5, 41]. It seems ironic that the lack of adequate resources prevent the development and implementation of AI applications in LMICs, where through the use of AI, healthcare could eventually become more cost-effective. This fact should be realized by funding and research agencies globally, so sufficient funds and resources could be directed towards for researchers in LMICs researchers to explore the various avenues of AI application in healthcare in these countries.

Although there is a burgeoning interest in the use of AI in dentistry, its translation from academic investigation to practical software application remains a work in progress. To better understand the deployment of AI in LMIC, we also stratified the included studies in our review according to the maturity of their development stages, as described by Zhang and colleagues [38]. This model describes four stages: math into algorithm, algorithm into model, model into device, and device into practice. Majority of research in AI in dentistry was focused on the algorithm-to-model stage, involving testing performance of models on labeled datasets. Only three studies progressed to the model-into-device stage. One such study, conducted by Mariam et al., used gaze-based annotation of histology images to compare the efficiency of AI versus human annotation finding AI to be more efficient [16].

The other two studies compared WebCeph, an online orthodontic software tool that uses AI technology for cephalometric analysis. Both studies utilized indigenous datasets and compared manual tracing with WebCeph tracing, but did not find any statistically significant differences in the tracing accuracy [32]. However, WebCeph was significantly faster. Notably, the authors of this review were unable to obtain details about the algorithm and dataset used to train the WebCeph model. Finally, one study utilized a machine learning algorithm to improve patient recall rate by 15% [29]. Different studies have been conducted to enhance the field of orthodontics, such as a study by Bahrami et al., emphasizing on Smart Orthodontic Brackets where utilization of machine learning algorithms can help improve and optimize orthodontic treatment outcomes [46].

Only two studies have attempted to validate the deployment of the device in a real-world environment. One study by Ehtesham et al. used AI as a decision support system for oral pathology detection. It is worth reporting that the deployment period lasted only 5 days, and the validation was conducted on a small sample of 39 patients [14].

Artificial intelligence (AI) holds promise in enhancing workflow and efficiency within healthcare practices. As such, it is essential that the AI system is user-friendly and compatible with existing infrastructure for effective implementation. However, this study had limitations, including a short duration of observation and limited number of patients, and was not entirely representative of the true environment for AI implementation and therefore warrants further research.

The other study by Ghosh et al. used bespoke messages, based on patients treatment needs, to send reminders for follow up visits which helped improve patient recall rate. This was the only study that described the patient experience with AI implementation in dental clinics [29].

In terms of future-orientated approach, there is an essential need to develop regulations concerning patient safety, confidentiality and privacy when AI is actually implemented in the real world settings. Moreover, there was a lack of cost-effectiveness or cost-utility analyses among the studies, highlighting a crucial gap in research that needs to be evaluated further. Besides the use of AI in clinical dentistry, its utility for academics and training of dental students is also an avenue worth exploring.

This study is unique since it attempts to holistically identify the research that has been conducted and subsequently published pertinent to the application of AI in dentistry hailing from LMICs. The authors of the included studies included did not mention any specific limitation in conducting AI based research in LMICs in terms of constraint of resources. However, the fact that only 25 studies from around the globe could be included in this review is a testament to the fact that the ongoing research in LMICs is significantly deficient. This could be attributed to the dearth of resources, among other possible factors which need to be further investigated. We found all included studies to have mentioned the deep learning techniques, algorithms, and Al model as well as datasets that they used in their research. However, another study done on the AI application in the healthcare sector in LMICs reported half of the included studies did not mention this information, even though the review focused on research done on real world application of AI [47].

Some limitations that were faced in conduction of this review included the difficulty in making specific and clear-cut conclusions from results of the included studies because of variability in study methodologies, reporting of the results, and the AI entities used. Therefore, a summary chart was included in order to display the results of the included studies.

## Conclusion

This scoping review identified all pertinent literature related to AI application and research in dentistry, hailing from LMICs. It was found that most studies were from India, done in the specialty of Orthodontics, focusing on diagnostic test accuracy with validation study design and a quantitative approach. Most frequent datasets used were OPGs, with limited number of datasets in all included studies. Majority of the studies reported AI maturity at level 2. There was no reporting of limitations that researchers faced based on the lack of resources in LMICs but the scant research available from LMICs in this domain is a clear indication of this. There was no mention of cost-utility analysis or patient experience, which are gaps that need to be addressed in subsequent studies. In addition, there was a great variability in reporting of results. Overall, it was seen from the limited number of studies included in this review that the utility and research relevant to AI in dentistry is significantly deficient in LMICs. With the interest and support of the global community, researchers in LMICs would be able to attain the necessary resources to expand the application and utility of AI in dentistry in these countries, resulting in a greater number of research publications.

### Supplementary Information

**Additional file 1.**

## Data Availability

All data generated or analyzed during this study are included in this published article [and its supplementary information files].
