# Supplementary material for: Research and application of artificial intelligence in dentistry from lower-middle income countries – a scoping review
Source: BMC Oral Health. 2024 Feb 12;24:220. doi: 10.1186/s12903-024-03970-y (PMC10860267; doi:10.1186/s12903-024-03970-y)
Supplement: Supplementary file 1 — Additional file 1. [file 12903_2024_3970_MOESM1_ESM.docx]

**Supplementary Tables**

**Table A: Characteristics of the included studies**

| **S.No** | **Study Characteristics** |  |
| --- | --- | --- |
| 1 | ***Region of Origin*** | **n (%)** |
|  | **Asian** | **16 (64%)** |
|  | India | 12 (48%) |
|  | Pakistan | 03 (12%) |
|  | Nepal | 01 (4%) |
|  | **Middle East** | **4 (16%)** |
|  | Egypt | 01 (4%) |
|  | Iran | 03 (12%) |
|  | **South East Asian** | **5 (20%)** |
|  | Indonesia | 02 (8%) |
|  | Vietnam | 03 (12%) |
| ***2.*** | ***Specialty*** | **n (%)** |
|  | Orthodontics | 07 (28%) |
|  | Endodontics | 06 (24%) |
|  | Restorative Dentistry | 04 (16%) |
|  | Oral and Maxillofacial surgery | 03 (12%) |
|  | Oral Pathology | 02 (8%) |
|  | Periodontics | 01 (4%) |
|  | Oral Radiology | 01 (4%) |
|  | Not specified | 01 (4%) |
| ***3.*** | ***Type of study*** | **n (%)** |
|  | Validation | 22 (88%) |
|  | Pilot | 01 (4%) |
|  | Randomized parallel group | 01 (4%) |
|  | Case control | 01(4%) |
| ***4.*** | ***Study methodology*** | **n (%)** |
|  | Quantitative | 20 (80%) |
|  | Qualitative | 02 (8%) |
|  | Mixed Method | 03 (12%) |
| ***5.*** | ***Study theme*** | **n (%)** |
|  | Diagnostic test accuracy | 14 (56%) |
|  | Prediction | 05 (20%) |
|  | Identification | 03 (12%) |
|  | Classification accuracy | 01 (4%) |
|  | Correlation | 01 (4%) |
|  | Dental office administration | 01 (4%) |
| ***6.*** | ***Source of Data set*** | **n (%)** |
|  | Local | 19 (76%) |
|  | Local and international | 01 (4%) |
|  | Others (internet sources, mobile camera) | 01 (4%) |
|  | Not specified | 04 (16%) |
| ***7.*** | ***Type of Data sets mentioned (overall)*** | **n** |
|  | OPGs | 08 |
|  | Lateral Cephalograms | 02 |
|  | Intraoral photographs | 01 |
|  | Health records | 03 |
|  | Clinical pictures | 02 |
|  | Dental clinical data | 04 |
|  | Periapical radiographs | 02 |
|  | Bitewing radiographs | 01 |
|  | Dental casts | 02 |
|  | Cystatin S levels of saliva | 01 |
|  | Histology patches | 01 |
|  | CBCT | 01 |
|  | Radiovisographs | 01 |
|  | AAE Case difficulty Assessment forms | 01 |
|  | Scientific papers | 01 |
| ***8.*** | ***Tasks accomplished/Issues addressed (overall)*** | **n** |
| ***a.*** | **Identification of structures** |  |
|  | Teeth detection and numbering | 01 |
|  | Length measurement/ apical foramen | 01 |
|  | Root morphology | 01 |
|  | C-shaped root canal anatomy | 01 |
|  | Implant system | 01 |
| ***b.*** | **Prediction of variables** |  |
|  | Gender | 03 |
|  | Case difficulty | 02 |
|  | Postoperative pain after implant placement | 01 |
| ***c.*** | **Detection of Pathologies/Anomalies** |  |
|  | Caries | 03 |
|  | Periapical lesions | 03 |
|  | Periodontitis | 01 |
|  | Malocclusion | 01 |
|  | Root fracture | 01 |
|  | Diabetes tongue | 01 |
| ***d.*** | **Others** |  |
|  | Orthodontic treatment planning | 01 |
|  | Cephalometric analysis | 01 |
|  | Decision support system for differential diagnosis | 01 |
|  | Anatomical risk factors related to number of traumatized teeth | 01 |
|  | Gaze pattern for annotation | 01 |
|  | Patient recall rate | 01 |

**Table B: Types of AI entities mentioned in the included studies**

| **1** | **Neuronal networks** |
| --- | --- |
|  | CNN |
|  | Dense Net |
|  | U-Net |
|  | Residual U-Net |
|  | Xception U-Net |
|  | VGG |
|  | DL (MDP (maximum directional pattern) using CNN |
|  | YOLO v3 with DarkNet53 architecture |
|  | YOLO v5 with DarkNet53 architecture |
|  | ANN |
|  | MLP model of ANN |
| **2.** | **Models** |
|  | Mask RCNN with back bone |
|  | Resnet 50 |
|  | Resnet 101 |
|  | RetinaNet |
|  | Mobile Net V2 |
|  | Regression |
|  | Topic modeling using Labeled LDA (Latent Dirichlet Allocation) |
|  | Faster Region-Based Convolutional Neural Networks (Faster R-CNNs) |
| **3.** | **Algorithms** |
|  | YOLO |
|  | Nearest Neighbor algorithm |
|  | XGBoost |
|  | Random Forest |
|  | SVM |
|  | XGB Classifier |
|  | Random Forest Classifier |
|  | Linear SVM |
|  | Logistic Regression |
|  | Decision Tree Classifier |
|  | K-Neighbor Classifier |
|  | Naive Bayes Classifier |
|  | ML (Multiple Linear Regression MLR) model |
|  | Deep neural network (DNN)) |

**Table C: Definitions of Artificial Intelligence and its types**

| Artificial intelligence (AI) | The term is applied when a computing device mimics human cognitive functions,such as learning and problem solving. |
| --- | --- |
| Machine Learning | A form of AI that learns information and patterns directly from data without being explicitly programmed. It  makes predictions on the basis of acquired data through an algorithm. |
| Neural network (NN)/(ANN) | Consists of ML algorithms called neurons (nodes) that focus on recognizing underlying patterns or  connections from a dataset and imitate the data processing function of human brain. |
| Deep learning (DL) | The “deep” in deep learning is referring to the depth of layers in a neural network. A neural network that  consists of more than 3 layers that would be inclusive of the inputs and the output can be considered a  deep learning algorithm. |
| Convolutional neural network (CNN) | A subset of deep learning suitable for processing visual and other two-dimensional data. |
